# Surgical Clinic at Harlem Hospital

**Published:** 1893-07

**Authors:** Thomas H. Manley

**Affiliations:** New York


ATLANTA
### MEDICAL AND SURGICAL JOURNAL.

VOL. X. JULY, 1893. No. 5.

EDITED BY
LUTHER B. GRANDY, M. D., AND MILLER B. HUTCHINS, M. D.,
WITH THE CO-OPERATION OF
H. V. M. MILLER, M. D., LL. D., VIRGIL O. HARDON, M. D.,
FLOYD W. McRAE, M. D., AND ARTHUR G. HOBBS, M. D.

### *CLINICAL LECTURE.*

### SURGICAL CLINIC AT HARLEM HOSPITAL.

### HARELIP, CLEFT THROUGH LIP, GUM, HARD AND SOFT PALATES. SARCOMA OF THE TESTES. DEPRESSED FRACTURE OF THE SKULL. THORACIC NEOPLASM.

BY THOMAS H. MANLEY, M. D.,
NEW YORK.

The first case on the list is one of unusual interest, and represents a most repulsive deformity, when of the aggravated type. The infant is six weeks old, the fourth child of healthy, well-formed parents.

There is no history of harelip on either side of the house, though their first-born was similarly deformed, but not in so severe a degree. That child was operated on at an early age—the third week—with excellent result, and is now a hardy, active boy of ten years. There is no history of fright on the part of the mother during pregnancy in this case, though it seems there was in the first. The infant has been unable to nurse, owing to the absence of a vacuum.

The deformity consists, as you see, of a complete cleft, extending through and widely separating the lip, the superior and inter-maxillary bones on the left side; and by the breach which it produces, making the mouth and left nasal canal one large, common cavity.

It will be observed that the intermaxillary bone is crowded far away from the median line, to the right, and with it is carried the nasal septum, so that the normal, triangular outline of the nostrils' aperture is lost; and instead nothing remains but a long transverse slip with a lateral collapse and turning in of the columnæ nasi.

Within the mouth the degree of separation through the palatine vault is very considerable.

With all these cases it is my invariable rule, when an opportunity offers, to operate at the earliest possible date.

My reasons for so proceeding are:

1. Because at this age the infant has no sense of pain.

2. The bones are plastic and are elastic.

3. After reposition is effected in the formative processes of evolution and growth, the tendency will be towards a greater degree of perfection as far as functions and æsthetic effects go, than when they are delayed to a later date. Of course if the infant were young and delicate it would be better to delay operation until a later date.

Probably there is no class of deformities of congenital or acquired origin for which modern surgery has done so much as in those of this description.

I may say in epitome, that in all operations for harelip there are a few simple principles to be applied and conditions observed, where the results will generally be most gratifying, and the mortality practically nothing. First, and of cardinal importance, is osteoclasis, *i. e.*, the reposition of the displaced bone and a filling in of gap; that this should be effectually reproduced is of vital importance, for it is practically the key to the whole operation. By this we restore the continuity of the alveolar arch, restore the lost functions of mastication, relieve the tension of the soft parts, so that their approximation will be easily effected, and reunion promptly accomplished. And further, it will restore harmony in symmetry and produce a most satisfactory æsthetic effect. The next is, sacrifice no parings or particle of tissue. Effective hæmostasis, thorough asepsis, and finally, but little anæsthetic.

I shall, having prepared the gums and crushed the dividing borders of the maxillary bones together, insert a heavy shotted silver wire, passing completely through the bone. This will be left in position for three or four weeks. You will notice, that instead of trimming the surface of the cleft, I split it. The inferior mucous border is severed separately. Now, light, shotted silver wire sutures, to the number of three, are introduced and fixed, after which a superficial layer of silk sutures are adjusted. It is of vast importance in these cases that they be efficiently nursed, and kept as quiet as possible after operation.

The next case is one in which the diagnosis of sarcoma of the testes has been made by the patient's physician. But on a rigorous examination it seems more like a hydrocele with some complicated elements. It is true, that the exploratory puncture would help to clear up confusion, but he would not consent to it without ether.

On examination, I find it of a stony hardness, extending from the testis along the inguinal canal as far as the internal ring. The mass evidently is not a hernia. It is painless, and the patient denies traumatism. He comes in only because of its bulk and weight, which interfere with his work.

On incision, it is seen that it is a large, sanguineous effusion into the tunica-vaginalis and the connective tissues. Here at the base, running transversely, is noticed a rent through the tunica-vaginalis and an escape into the adjacent tissues. In this connection it might be interesting to inquire how the serous envelope became ruptured; whether by violence, or by the sac giving way, through over tension and pressure, while asleep.

He denies traumatism. But, like many of our dissipated hospital patients, his word is not to be relied on. In this case, the transmitted light test was negative, because of the sanguineous effusion and black, pigmentary deposit, which lined the wall of the serosa.

Our next case for operation, as with the one just returned to the ward, has a mass which presents many puzzling clinical and physical characteristics.

Patient, a man, of good general health, twenty-six years, with no cachexia and no fever; presents a large, flattened, insensitive fullness, which is submammary and mainly occupies the præcardia. He says he never was injured here, and that his health is good. The tumor, which is painless, has been growing, he tells us, six months. Latterly, its apex has been inflamed and inclines to point. After a process of exclusion, we have decided that the mass was probably a lipoma undergoing central suppurative degeneration.

As we make an exploratory incision, however, we find an inky black, clotted material issue through in great quantities.

After scooping away a large amount of broken down material, we find a large cavity, at the bottom of which we come on to three fractured ribs to the left of their sternal third. Their shafts are softened, bloodless and necrotic for some distance on either side of the neoplasm.

Immediately under the flow of this formation, the heart's impulse could be readily defined. And by carrying the finger a little inward toward the sternum, the internal mammary artery can be readily felt.

We have now gouged away a quantity of the osteal structures, but we have not opened the pleura.

What have we here?

Is it a case of melanotic cancer?

Well! the microscope will definitely decide that question.

But, after all, may not all this have come from an injury? He admits being a hard drinker. My impression is, that it is the latter.

The next and last case, is one of fracture of the skull. Another unfortunate, and though seventy-two years old, his drinking habits are yet in the ascendency. He was picked up by the police and sent in on an ambulance last night. He has a fracture immediately between, but a little below the glabella; or, in other words, practically into the frontal-sinus.

Since he came in, he has had delirium tremens. He has no fever and no cerebral symptoms, from which we must infer that only the outer table is fractured.

You will observe that we give him no ether, but simply reopen the wound. To dispense with pulmonary anæsthetics is a great gain to the patient on whom we do cranial-surgery. It will be noticed, too, for removing the depressed outer table, that we use no trephine. I may say that the fine, strong grooved chisel offers a thousand advantages over the trephine, and greatly simplifies what was formerly always a complicated and dangerous operation. You see here that these fragments are reunited with the greatest ease with the chisel or osteo-joint. The inner table is not fractured. The old man will soon be well and out again.

